# Air pollution and indoor settings

**DOI:** 10.1016/j.waojou.2020.100499

**Published:** 2021-01-07

**Authors:** Nelson Augusto Rosário Filho, Marilyn Urrutia-Pereira, Gennaro D'Amato, Lorenzo Cecchi, Ignacio J. Ansotegui, Carmen Galán, Anna Pomés, Margarita Murrieta-Aguttes, Luis Caraballo, Philip Rouadi, Herberto J. Chong-Neto, David B. Peden

**Affiliations:** a University of Parana, Curitiba, PR, Brazil; b Department of Medicine, Universidade Federal Do Pampa, Bagé, RS, Brazil; c Division of Respiratory and Allergic Diseases, High Specialty Hospital A. Cardarelli, School of Specialization in Respiratory Diseases, Federico II University, Naples, Italy; d Centre of Bioclimatology, University of Florence, Florence, Italy; SOS Allergy and Clinical Immunology, USL Toscana Centro Prato, Italy; e Hospital Quironsalud Bizkaia, Bilbao-Erandio, Spain; f Department of Botany, Ecology and Plant Physiology, International Campus of Excellence on Agrifood (ceiA3), University of Córdoba, Córdoba, Spain; g Basic Research, Indoor Biotechnologies, Inc, Charlottesville, VA, United States; h Consumer Healthcare Division, Sanofi-Aventis Group, Gentility, France; i Institute for Immunological Research, University of Cartagena, Cartagena, Colombia; j Department of Otolaryngology- Head and Neck Surgery, Eye and Ear University Hospital, Beirut, Lebanon; k Division of Allergy and Immunology, Department of Pediatrics, Federal University of Paraná, Curitiba, PR, Brazil; l UNC School of Medicine, University of North Carolina, Chapel Hill, NC, United States

**Keywords:** Pollution, Environmental pollution, Indoor air pollution, Air pollutants, AR, allergic rhinitis, BAL, bronchoalveolar lavage, CO, carbon monoxide, CO2, carbon dioxide, COPD, chronic obstructive pulmonary disease, DEPs, diesel exhaust particles, FeNO, fractional exhaled nitric oxide, FEV1, forced expiratory volume, GM-CSF, granulocyte and macrophage growth stimulating factor, GST, glutathione S-transferase, HAP, household air pollution, HEPA, High Efficiency Particulate Arrestance, ILC2, innate lymphoid cells, NCD, non-communicable disease, NO, nitric oxide, NO_2_, nitrogen dioxide, O_3_, ozone, PM, particulate matter, PMNs, polymorphonuclear leukocytes, SO_2_, sulfur dioxide, TRAP, Traffic-related air pollution, TSLP, thymic stromal lymphopoietin, VOCs, volatile organic compounds, PAH, polycyclic aromatic hydrocarbons

## Abstract

Indoor environments contribute significantly to total human exposure to air pollutants, as people spend most of their time indoors. Household air pollution (HAP) resulting from cooking with polluting (“dirty”) fuels, which include coal, kerosene, and biomass (wood, charcoal, crop residues, and animal manure) is a global environmental health problem. Indoor pollutants are gases, particulates, toxins, and microorganisms among others, that can have an impact especially on the health of children and adults through a combination of different mechanisms on oxidative stress and gene activation, epigenetic, cellular, and immunological systems. Air pollution is a major risk factor and contributor to morbidity and mortality from major chronic diseases. Children are significantly affected by the impact of the environment due to biological immaturity, prenatal and postnatal lung development. Poor air quality has been related to an increased prevalence of clinical manifestations of allergic asthma and rhinitis. Health professionals should increase their role in managing the exposure of children and adults to air pollution with better methods of care, prevention, and collective action. Interventions to reduce household pollutants may promote health and can be achieved with education, community, and health professional involvement.

## Background

Indoor air pollution encompasses diverse biological contaminants such as allergens, mainly house-dust mites, as well as insects, pollen and animal sources, molds, and bacterial endotoxins. Other components are chemical air pollutants, such as gases, particulate matter, formaldehyde, and volatile organic compounds (VOCs).^1^ This paper is focused only on non-biological indoor pollutants and their effects on human health and is not intended to be an exhaustive review on the subject.

Household air pollution (HAP) resulting from cooking with polluting (“dirty”) fuels, which include coal, kerosene, and biomass (wood, charcoal, crop residues, and animal manure) is a global environmental health problem. It affects approximately 2.45 billion people in developing countries.^1^^,^^2^ This type of pollution causes between 2.8 and 4.3 million premature deaths each year, equivalent to 7.7% of global mortality, which is more than the burden of malaria, tuberculosis, and HIV/AIDS combined.^3^

Of these premature deaths, 3.8 million are caused by non-communicable diseases (NCDs). Among those NCDs, HAP is estimated to cause 25% of all stroke deaths, 15% of ischemic heart disease deaths, 17% of lung cancer deaths, and more than 33% of all deaths from chronic obstructive pulmonary disease (COPD).^4^ HAP is the second largest risk factor for women and girls; and for men it is the fifth largest risk factor, after smoking, alcohol use, and high blood pressure.^4^

Although smoking is the leading cause of COPD in the world, exposure to HAP is likely to be a preventable factor in low- and middle-income countries, especially in women. Children from low-income families are at high risk for suffering from the diseases associated with this contaminant, due to their greater involvement in domestic activities with their mothers as well as still having an immature immune system. They are exposed to smoke concentrations at levels well above that recommended by the World Health Organization (WHO).^4^ Children are also at risk due to increased respiratory rates, proximity to the floor/settled dust, actively developing lung, and airway tissue.

Indoor pollutants derive mainly from human activity at home and in schools, but also in day-care centers, social recreation settings, or microenvironments, such as cars, buses, trains, and aeroplanes.^1^ It is estimated that air pollution will cause the death of 800,000 people from COPD and 280,000 people from lung cancer.^4^ There is an overlap in the 2 forms of pollution consequences and in the 2 diseases. Most of the literature addressing attributable mortality to air pollution is based on outdoor measures of pollutants. Nevertheless, indoor air quality is influenced by penetrating outdoor air that sums up to specific indoor pollution sources, interactions between building system/construction techniques, and occupants.^5^

Many different pollutants, such as ozone (O_3_,), particulate matter (PM), sulfur dioxide (SO_2_) and nitorgen dioxide (NO_2_), have irritating effects that can induce cough, increased mucus, and bronchial hyperresponsiveness, which result in a greater number of visits to the emergency department for asthma, COPD, and respiratory symptoms that are often attributed to respiratory infections.^1^, ^2^, ^3^, ^4^

**Fig. 1:**
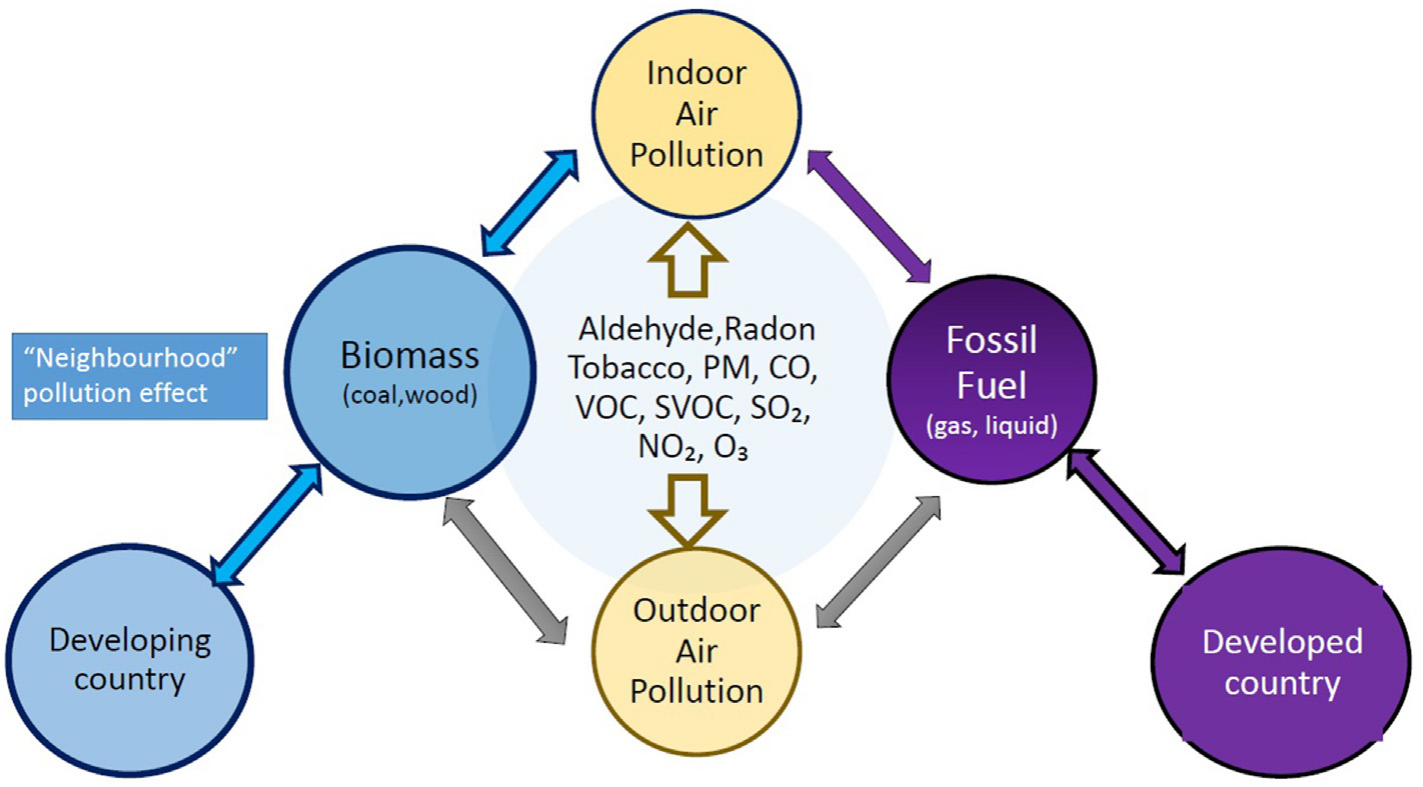
Indoor air pollution – sources and Geo-epidemiological correlates. Indoor air and outdoor air share basically the same pollutants but in different proportions. In densely populated areas using biomass, indoor air pollutants may become outdoor—the so called “neighbourhood” pollution effect.

Indoor air and outdoor air share the same pollutants but in different proportions. In densely populated areas using biomass, indoor air pollutants may become outdoor—the so called “neighbourhood” pollution effect (Fig. 1).

## Mechanisms

The pathogenesis of household pollution results from a combination of effects at different levels. Most of the literature addressing attributable mortality to air pollution is based on outdoor measures of pollutants and should be taken into consideration.

### Effects of gases

The damage to human tissue by polluting gases depends on their solubility in water, concentration, ability to oxidize tissues, and the susceptibility of the affected person. SO_2_ is highly soluble in water, and to a large extent damages the upper airways and the skin, while NO_2_ and O_3_ are less soluble and, therefore, can penetrate more deeply into the lung.^1^

The commonly used indoor ozone emission devices include photocopyiers, room air purifiers, disinfecting devices, and laser printers.^6^ In a study, indoor ozone concentration fluctuated between 20% and 80% of the outdoor ozone concentration depending on the air exchange rate.^7^

Carbon monoxide (CO) and nitric oxide (NO) are highly soluble and non-irritating and passes quickly into the bloodstream; their toxicity result mainly from competing successfully with oxygen for binding to hemoglobin, which results in tissue hypoxia.^1^^,^^8^

### Effects of particulate matter

Due to the extensive evidence of the health effects, the average daily and annual concentrations of air particulate matter, PM_10_ and PM_2.5_, are regulated in accordance with the World Health Organization (WHO) Air Quality Guidelines and standards in several countries. The fine particles (PM_2.5_) enter the pulmonary alveoli, and are readily captured by cells and transported by the bloodstream; the ultrafine particles (PM_0.1_) pass easily through the alveolar-capillary membrane and, therefore, have the greatest systemic toxicity.^8^, ^9^, ^10^

Toxic components may be present on the surface of the particles and be responsible for tissue damage upon contact. Thus, elements such as arsenic, lead, or cadmium, or compounds such as sulfuric acid or polycyclic aromatic hydrocarbons, can be inhaled during the combustion process that generates PM and transported deep into the lung on the surface of ultrafine particles.^1^^,^^10^

This situation is more relevant for particles resulting from the combustion of fossil fuels, especially the combustion of coal, which contains many heavy metals and high levels of sulfur, and the burning of oil derivatives responsible for the formation of the discharge particles of diesel (DEPs). Thus, exposure to pollutants, including toxic metals, organic compounds, and gases, in addition to invading an organs and causing direct damage, can cause increased levels of markers of airway inflammation (eg, polymorphonuclear leukocytes [PMNs] or inflammatory cytokines in bronchoalveolar lavage [BAL] or sputum, and increased fractional exhaled nitric oxide [FeNO] in asthmatics) with multisystem effects.^8^

The lung faces harmful effects of filtering PM, overloading the macrophage function and lymphatic system, leaving deposits of material centered around the terminal bronchioles and first-generation respiratory bronchioles. PM can lead to chronic local inflammation and fibrosis and predispose to lung carcinoma. Repeated insults of pollution can also contribute to diabetes mellitus and cardiovascular conditions such as atherosclerosis, with a wide range of effects on lipid metabolism and oxidative stress.^1^^,^^9^

### Effects on oxidative stress and gene activation

Exposure of respiratory epithelium to particulate pollutants and DEPs triggers oxidative stress pathways and activates oxidative stress response genes. Depletion of antioxidants and pro-inflammatory signaling occurs, such as the synthesis of cytokines and mediators that trigger a cascade of events that can affect distant organs. The greater the surface area of the ultrafine particles, the greater is their ability to produce oxidative stress.^9^, ^10^

The response to oxidative stress and the immune and inflammatory responses to air pollutants can be genetically regulated. Several genes are involved in inflammation and control of synthesis of glutathione *S*-transferase (GST), an intracellular enzyme with an anti-oxidant effect. Polymorphisms in the GST gene can modify the impact of the response to pollution on respiratory and allergic diseases.^11^ Individuals with certain GST genotypes would be more susceptible to domestic exposure, with a higher risk of asthma and deficit in lung function.^12^^,^^13^

### Epigenetic effects

Prenatal exposure to NO_2_ is associated with DNA methylation in several genes related to mitochondria, as well as in several genes involved in antioxidant defense pathways.^12^, ^13^, ^14^, ^15^, ^16^, ^17^ Epigenetics modulate genetic and physiological responses to air pollution, being an important cause of susceptibility to health effects related to pollution. For example, epigenetic changes including DNA methylation, histone modifications, and uncoded RNAs have been considered important additional mechanisms in the development of asthma and its impressive increase in prevalence in recent decades, highly influenced by environmental exposures.^7^^,^^15^^,^^17^ The exposure to specific environmental factors can be a key factor in the induction or suppression of asthma-related genes. Also, epigenetic changes in the respiratory epithelium may occur due to respiratory infections, exposure to pollutants like PM_2.5_, and maternal smoking.^15^^,^^16^

### Cellular and immunological effects

Increased exposure to PM is associated with an increase in serum biomarkers such as C-reactive protein, fibrinogen, leukocytes, blood platelets, plasma viscosity, adhesion molecules, and a set of cytokines and inflammatory mediators that overload the endothelium, causing the loss of its modulator function.^16^^,^^17^

Exposure to PM and some polluting gases modulates the respiratory epithelium and promotes the production of several T-2 profile cytokines, including IL-1, IL-6, IL-8, IL-25, IL-33, TNF alpha, TSLP, and GM-CSF.^18^^,^^19^ Several studies have shown an increase in TSLP, IL-25, and IL-33 in the airways of asthmatics, and this increase is related to the severity of the disease.^20^ Therefore, the oxidative stress of respiratory epithelial cells depends on several factors (healthy vs allergic, nose vs lung, nature of pollutant) resulting in the induction of an immune response with a Type-2 profile, which would be associated with worsening asthma.^9^^,^^21^^,^^22^

Cultured nasal epithelial cells exposed i*n vitro* to PM_2.5_ may lose the barrier function through decreased expression of tight junction proteins and increased release of proinflammatory cytokines IL-8, TIMP metallopeptidase inhibitor 1, and TSLP.^23^ These could lead to susceptibility to rhinitis and rhinosinusitis in highly PM_2.5_ polluted areas.^24^

The cytokines derived from the epithelium, IL-25, IL-33, and TSLP, in response to oxidative injury resulting from exposure to pollutants, can induce the activation of Type 2 innate lymphoid cells (ILC2), which are capable of producing large amounts of Th2 cytokines such as IL-13, IL-5, and IL-9.^25^^,^^26^ The action of IL-5 and IL-13 causes eosinophilia and bronchial hyperreactivity; this mechanism is associated with the asthma Type-2 high endotype and does not depend on antigenic stimulation.^22^ This sequence of events could explain the association between the increase in pollution and the increase in the prevalence of childhood asthma observed in recent years.

## Pre-natal and post-natal influences of air pollution on children

Data published by the WHO show that air pollution has a wide and dreadful impact on children's health and survival. The negative effects of climate change are widespread; 1 in 4 deaths of children under 5 years of age is directly or indirectly related to environmental risks. Both ambient and home air pollution contribute to respiratory tract infections, which in 2016 caused 543 000 deaths of children under 5 years of age related to environmental risks.^27^

Children are significantly affected by the impact of the environment due to: biological immaturity; prenatal and postnatal lung development; higher energy and metabolic consumption; social behavior such as crawling, bringing objects to their mouths, becoming more exposed to potential contaminants of dust, soil, toy components, or household cleaning products; higher life expectancy; and having no decision-making capacity on environmental issues.^28^^,^^7^ Prenatally, it has been shown that exposure to ambient air pollution begins to influence the uterus and may impair placental development, causing a decrease in placental size in oxygen and nutrient transport, placental aging, oxidative stress, and, changes in telomere length and mitochondrial content.^29^ Bové et al reported the presence of black carbon (BC) particles as part of combustion-derived particulate matter in human placenta. Black carbon particles, accumulated on the fetal side of the placenta, which suggests that they can be transported to the fetus, representing a potential mechanism that would explain the harmful health effects of pollution since early life.^30^ The maternal environment and exposures can alter fetal lung development, predisposing to deterioration in lung development, lung function, and future respiratory diseases.^27^

Exposure to NO_2_, PM_2.5_, and PM_10_ during pregnancy and during the first year of life was associated with the higher prevalence of abnormal lung function and development of asthma in children.^31^, ^32^, ^33^

The longest longitudinal study of the health of school-aged children in Canada with more than 17 years of follow-up have demonstrated that exposures to oxidant air pollutants (O_3_ and NO_2_) but not PM_2.5_ were associated with an increased risk of incident asthma (17%) and eczema (7%) in children.^34^

The Lancet Countdown's report on the impact of climate change on children's health warns us through published indicators that we are living in a world struggling to cope with the impact of global warming which occurs faster than governments are able or willing to handle.^35^ Increasingly, indoor pollutant levels are worrying for children in the inner city and in developing countries.^36^, ^37^, ^38^, ^39^

Given that children spend a long time in school buildings, we can predict that the conditions in such buildings may predispose to respiratory diseases.^40^ Several pollutants, such as bacteria, molds, VOCs, CO, CO_2_, NO_2_, PAH, and PM, have been found in classrooms.^41^^,^^42^

Exposure to NO_2_ in the school classroom microenvironment is significantly related to airflow limitation in children with asthma through a pathway that is not dependent on allergic inflammation.^41^

Schools usually lack sources of cooking, have central locations in community, prohibit smoking, and have uses and cars idling outside. Traffic pollution is an important source of exposure in the school because many schools are close to a major roadway.^43^ School children are exposed to high concentrations of air pollutants in the classroom as defined by the WHO for PM_2.5_ and NO_2_.^44^

Associations have been reported between the concentrations of pollutants and the onset of health problems in schoolchildren, mainly respiratory/allergic symptoms and diseases.^41^

The schools in urban areas reveal high exposure to particulate matter from the highways of motor vehicles, indicating the anthropogenic sources near to schools. For suburban areas, the analysis indicates the proximity with anthropogenic sources, especially the nearby industrial plants. These outdoor sources impact indoor air quality in both homes and schools.^42^

Contributing factors to indoor air quality include: classroom occupancy rates, building age, winter months and climate conditions, classroom ventilation, indoor activities, the degree of outdoor air pollution, and sources of indoor pollutants.^45^, ^46^, ^47^ High CO_2_ concentrations in classrooms, which indicate poor ventilation, and the increasing PM in urban outdoor air have been identified as primary causes of poor indoor air quality in schools.

Bowatte et al reported that exposure to traffic-related air pollution (TRAP) is associated with increased risk of sensitization to common allergens during childhood whereas the larger, more standardized multicenter study that included data from 5 birth cohorts found no clear associations.^48^

The subject had further input by Fuertes and Heinrich that claimed consistent evidence was needed to clearly show that exposure to TRAP is associated with increased risk of sensitization to common allergens during childhood.^49^

Only a few cohort studies have evaluated effects of exposure to air pollution on the development of sensitization in young children. In the birth cohort BAMSE (Children, Allergy, Milieu, Stockholm, Epidemiological Survey) there were no clear associations between estimated ambient exposure to traffic-related air pollution and overall sensitization to common inhalant and food allergens at 8 years of age.^50^

A prospective birth cohort study during the first 6 years of life in Germany (GINI Study) of approximately 3000 children, provided strong evidence for increased risk of atopic diseases and allergic sensitization when children are exposed to ambient particulate matter PM_2.5_, whereas no association was found for long-term exposure to NO_2_ and allergic sensitization.^51^

To et al examined associations between early life exposures to air pollution and incidence of asthma, allergic rhinitis, and eczema from birth through adolescence. Exposures to oxidant air pollutants (O_3_ and NO_2_) but not PM_2.5_ were associated with an increased risk of incident asthma (17%) and eczema (7%) in children.^34^In France, air quality in classrooms was poor, varying significantly among schools and cities. In a random sample, air quality was related to an increased prevalence of clinical manifestations of asthma and rhinitis in schoolchildren. Children with a background of allergies seemed at increased risk.^44^

A growing body of evidence shows that components of air pollution interact with airborne allergens and enhance the risk of atopic sensitization and exacerbation and development of symptoms in sensitized subjects.^46^

## Pollution effects on adult health

### Risk assessment

The concentration of PM_2.5_ is an important metric to evaluate HAP risk, although studies have demonstrated significant variation in HAP-PM_2.5_ concentrations at household, community, and country levels. The global risk due to HAP exposure is needed, as financial and resource constraints render it difficult to monitor exposures in all relevant areas.^52^

A Bayesian, hierarchical HAP- PM_2.5_ global exposure model was developed using female HAP- PM_2.5_ kitchen exposure in peer-reviewed studies from an updated WHO Global HAP database. This approach generates unique HAP- PM_2.5_ kitchen concentrations and personal exposure estimates for all countries, including those with little to no available quantitative HAP-PM_2.5_ exposure data. The global exposure model can add specificity and reduce exposure misclassification to enable an improved global HAP risk assessment.^52^

The 10-year Finnish Indoor Air and Health Programme develops operating models and guidelines for assessing the significance of indoor air quality problems in buildings at workplaces, homes, schools, and day care centers, and supports their systematic adoption. The programme focuses on the promotion of human health and well-being, the prevention of hazards, and improved communication as well as engages the whole health-care sector to manage patients' symptoms and complaints.^53^

### Cardiovascular

Air pollution has been strongly associated with an increased risk of mortality from cardiovascular disease, myocardial infarction, stroke, and hospitalization for congestive heart failure and hypertension.^54^^,^^6^ Mild increases in carboxyhemoglobin levels (by 3%–6%) occur when individuals are exposed to traffic pollution, and they can trigger angina and arrhythmias in individuals with coronary heart disease.^55^ Long-term exposure increases the risk of cardiovascular mortality and reduces life expectancy in more exposed segments of the population from several months to a few years.^56^ The overall evidence is consistent with a causal relationship between PM_2.5_ exposure and cardiovascular morbidity and mortality. Indoor exposure contributes to the increased risk of cardiovascular mortality.^56^

### Ocular

Conjunctivitis is associated with exposure to O_3_ and NO_2_, although PM_10_ and SO_2_ are also correlated. Lacrimal and eye irritation can occur as a reaction to visible fog, and this finding is often worse for contact lens wearers. Cataract formation has been described in women exposed to domestic air pollution in low-income countries.^12^ O_3_ levels and decreased humidity have been associated with dry eye disease, and air pollution, specifically PM and CO, has been associated with acute worsening of blepharitis.^55^^,^^57^

### Cancer

Atmospheric pollution has been classified as carcinogenic to humans by the International Agency for Research on Cancer based on evidence from epidemiological and animal studies. The levels of NO_2_ and O_3_ have been experimentally associated with cellular changes related to neoplasia: alteration in the length of telomeres and expression of genes involved in DNA damage and repair. Exhaustion of diesel engines was identified by the WHO as carcinogenic, based on evidence of a link with lung, colorectal, gastric, renal, and bladder cancer.^55^

### Skin

Several biological parameters that affect skin quality are influenced by pollution; cooking with solid fuels was associated with 5%–8% of more intense wrinkles on the face and with a 74% increased risk of fine wrinkles on the dorsal surface of the hands, regardless of age and other influences on skin aging.^58^

### Respiratory

HAP exposure has been associated with acute respiratory infections, tuberculosis, asthma, COPD, and pneumoconiosis.^59^ More studies with consistent diagnostic criteria and exposure monitoring are needed to accurately document the association between HAP exposure and respiratory diseases.^60^^,^^61^ Better environmental exposure monitoring is critical to better separate the contributions of HAP from that of other exposures, including ambient air pollution and tobacco smoking.^62^^,^^1^ One meta-analysis aimed to determine the association between indoor air pollution and risk of COPD. The eligible studies, case-control, retrospective cohort, and cross-sectional studies, were conducted in adults and assessed COPD using any diagnostic criteria.^63^ The results indicated that exposure to indoor air pollution due to biomass smoke is strongly associated with COPD.^63^

### Airway allergy

A review assessed the effects of indoor air pollution, outdoor air pollution, and subsequent climate change on asthma and allergic rhinitis (AR). The authors concluded that exposure to internal and external allergens is a well-established risk factor for the development of AR and asthma inducing more severe airway allergy phenotypes.^63^ Air pollution is associated with increased symptoms of sleep apnea, possibly because of inflammation of the upper airways by irritating pollutants and airborne allergens.

An in-depth analysis of the impact of pollutant exposure on AR has been published by the World Allergy Organization (WAO).^64^ Human experimental studies involving specific pollutant exposure and allergen challenge suggest pollution can exacerbate allergic airway disease and increase nasal responsiveness.^48^ Clinical and epidemiological studies demonstrate the immunological results from both aeroallergen and pollutant co-exposure.^25^^,^^64^^,^ Altered innate immunity (Toll like receptor signaling, Inflammasome activation) and altered adaptive immunity (allergy,IL-17 mediation, T cell regulation) mediate the risks of air pollution exposure and allergy.^34^^,^^65^^,^^66^

Air pollution can enhance T helper lymphocyte type 2 (Th2) and T helper lymphocyte type 17 (Th17) adaptive immune responses, as seen in allergy and asthma, and dysregulate anti-viral immune responses. An in-depth understanding of the immunological effects of ambient pollutants should hopefully yield new ideas on how to reduce the adverse health effects of air pollution.^67^

### COVID-19

Air pollution is a major risk factor for and contributor to the major chronic diseases that increase the severity and risk of death from COVID-19. High levels of air pollution affect the natural defenses of the body against airborne viruses, making people more likely to contract viral diseases, and this is likely to be true for SARS-CoV-2 as well. A clear correlation of COVID-19 infection in China with pollution levels and local climatic conditions with low temperature, mild daytime temperature range, and low humidity that would favor the transmission of the virus, has been demonstrated.^68^

This impact is highlighted by a study on COVID-19 deaths in the United States, indicating a dramatically increased risk of death in areas with higher average PM_2.5_ pollution levels in the past.^69^ COVID-19 mortality in Northern Italy, including Lombardy, Veneto, and Emília Romagna, was highly associated with the high levels of pollutants in these regions.^70^^,^^71^

This is the first preliminary evidence that SARS-CoV-2 RNA can be present on outdoor particulate matter, thus suggesting that atmospheric stability and high concentrations of PM could enhance the persistence of the virus in the atmosphere.^71^

However, the change in human behavior that resulted from the COVID-19 pandemic has demonstrated that major public health benefits and quality of life improvement can occur after reducing air pollution levels over just 1 month. A decrease in environmental pollution was demonstrated in Barcelona (Spain), after 2 weeks of quarantine due to the pandemic by the new coronavirus.^72^ The measures to combat the coronavirus have led to an approximately 40% reduction in average level of NO_2_ pollution and 10% reduction in average level of PM pollution over the past 30 days, resulting in 11 000 avoided deaths from air pollution (95% IC: 7000–21 000).^73^

## Benefits of reducing indoor air pollution

Household interventions in low- and middle-income countries have included: “clean” cook stoves with improved combustion efficiency or ventilation; the replacement of wood or kerosene by less-polluting fuels, such as ethanol or liquefied petroleum gas; and the use of air filters.^10^ Some, but not all, studies suggest that improving cook stove efficiency or venting emissions may be associated with reduced respiratory symptoms, lung function decline in women, and severe pneumonia in children.^74^ Several governments have initiated major programmes to accelerate the upgrade from solid fuels to clean fuels, particularly liquid petroleum gas, which provides research opportunities for the respiratory health community.^75^ Studies in high-income countries similarly involved fuel and stove switching as well as use of household air filters.^76^ All studies have shown that “clean” household air improves respiratory and non-respiratory symptoms, such as eye discomfort and headache.^77^

Interventions to improve kitchen ventilation and promote the use of clean fuels, installation of chimneys in households using coal stoves, improved cook stoves, and combustion efficiency have reduced the incidence of COPD and promoted better lung growth in children and slower decline in forced expiratory volume (FEV_1_) over time.^78^, ^79^, ^80^ Installing less-polluting heating (eg, heat pump, wood pellet burner, and flued gas) in the homes of children with asthma in New Zealand reduced symptoms of asthma, days off school, and healthcare use.^81^

Schools using unflued gas heating in winter were randomly allocated either to retain their heaters or to have replacement flued gas or electric heaters installed. Asthma attacks were significantly reduced in the intervention group.^82^ Thermal comfort, adequate ventilation to remove classroom-generated pollution, and the supply of filtered outdoor air are simple measures to improve air quality.^44^

Home-based intervention with air filters, including HEPA purifiers, found a reduction in asthma morbidity, and it may have other beneficial effects in nasal symptoms in children with AR.^83^^,^^84^ The long-term filtration of pollutants with an air conditioner filter was associated with cardiovascular health of adults and to decrease blood pressure.^57^ Research gaps and opportunities for interventions to reduce effects of solid fuel smoke on public health are well identified.^85^

## Recommended measures

Health professionals are reliable sources of information and advice; they play a very important role not only in treating health problems caused by air pollution, but also in educating family members and patients about risks and solutions, as well as in communicating with the general public and government leaders.^1^^,^^10^^,^^86^ (Table 1).

**Table 1:** Recommendations to health professionals on indoor pollution

Information: risk factors, identify sources of pollution
Investigate, publish, and disseminate knowledge
Prescribe solutions
Educate families and communities
Train other people
Advise on solutions for political representatives and leaders
Support better standards and policies to reduce harmful exposure
Advocate for monitoring
Emphasize the need to protect vulnerable people.

Health professionals should increase their role in managing the exposure of children and adults to air pollution with better methods of care, prevention, and collective action. Thus, they should:

• Be informed: Understand air pollution as a risk factor for people; identify the sources of environmental exposure in the communities where they work.
• Recognize medical conditions associated or related to exposure: A healthcare professional can identify risk factors related to air pollution by asking pertinent questions about the environments where their patients live or work.
• Investigate, publish, and disseminate knowledge: Health professionals can conduct research on the effects of air pollution on health and publish the results of studies on the causes, mechanisms, and effects of environmental exposure, as well as possible treatments, prevention, and management, and develop communication strategies for social and behavioral changes
• Prescribe solutions and educate families and communities: For problems related to air pollution, such as the consumption of fuels and appliances that consume less energy, health professionals can play a role.
• Training other people in the field of health and education: Health professionals can and should increase the scope of their messages about the health risks of air pollution and the strategies to reduce it. They can involve their colleagues in their workplaces, at local health centers, at conferences, and in professional associations. They can support the inclusion of children's environmental health in the curriculum of primary and higher education institutions, particularly in medical and nursing schools.
• Advise on solutions for political representatives and leaders from other sectors: Health professionals are well positioned to share their knowledge with decision makers, including members of local governments and school boards, and with other community leaders. Health professionals can faithfully convey the health burden caused by air pollution to leaders, support better standards and policies to reduce harmful exposure, advocate for monitoring, and emphasize the need to protect vulnerable people.

## Conclusion

Approaches to avoid exposure must be complementary and mutually reinforcing, at all levels: homes, schools, clinics, health institutions, municipalities, national governments, and the global community. Health professionals can come together to get leaders to take strong measures to protect the most vulnerable citizens, children who have little or no control over the air they breathe.

## Availability of data and materials

Not applicable.

## Disclosure information/Financial Support

Not applicable.

## Author contributions

M. Urrutia-Pereira made substantial contributions to the conception and design of the study, acquisition of data, analysis and interpretation of data, drafting the article, and revising it critically for important intellectual content. N.A. Rosário Filho, L. Caraballo, G. D'Amato, I.J. Ansotegui, and M. Murrieta-Aguttes made substancial contributions to the conception and design of the study, drafting the article, and revising it critically for important intellectual content. H.J. Chong-Neto, C. Galán, A. Pomés, and P. Rouadi made substantial contributions to the conception and design of the study, analysis and interpretation of data, drafting the article, and revising it critically for important intellectual content. L. Cecchi made substancial contributions to the conception and design of the study and revising the article critically for important intellectual content. D.B. Peden made substantial contributions to the drafting and revising the article.

## Ethics statement

Not applicable, as this is a review paper.

## Consent for publication

All authors reviewed the final version of the paper and agreed in writing to its publication in the World Allergy Organization Journal, as an open access article.

## Human and animal research

Not applicable, as this is a review paper.

## Registration of clinical trials

Not applicable, as this is a review paper.

## Declaration of competing interest

Dr. Ansotegui reports personal fees Mundipharma, Roxall, Sanofi, MSD, Faes Farma, Hikma, UCB, Astra Zeneca, Stallergenes, Abbott, and Bial, outside the submitted work.

Dr. Murrieta-Aguttes reports personal fees from Sanofi, outside the submitted work, and she is an employee of Sanofi-Aventis Group, Consumer Healthcare Division, France.

Dr. Pomés reports grants from NIH/NIAID, during the conduct of the study; other from Indoor Biotechnologies, Inc., outside the submitted work.

Dr. Caraballo, Dr. Cecchi, Dr. Chong Neto, Dr. D'Amato, Dr. Galán, Dr. Peden, Dr. Rosário, Dr. Rouadi, and Dr. Urrutia Pereira have nothing to disclose.
